# Reconciling the father tongue and mother tongue hypotheses in Indo-European populations

**DOI:** 10.1093/nsr/nwy083

**Published:** 2018-08-20

**Authors:** Menghan Zhang, Hong-Xiang Zheng, Shi Yan, Li Jin

**Affiliations:** 1 State Key Laboratory of Genetic Engineering and Collaborative Innovation Center for Genetics and Development, School of Life Sciences, Fudan University, Shanghai 200438, China; 2 Human Phenome Institute, Fudan University, Shanghai 200438, China; 3 Ministry of Education Key Laboratory of Contemporary Anthropology, School of Life Sciences, Fudan University, Shanghai 200438, China; 4 Chinese Academy of Sciences Key Laboratory of Computational Biology, CAS-MPG Partner Institute for Computational Biology, SIBS, CAS, Shanghai 200031, China

**Keywords:** Indo-European populations, Y-chromosomal haplogroup, mitochondrial DNA haplogroup, lexical system, phonemic system

## Abstract

In opposition to the mother tongue hypothesis, the father tongue hypothesis states that humans tend to speak their fathers’ language, based on a stronger correlation of languages to paternal lineages (Y-chromosome) than to maternal lineages (mitochondria). To reassess these two competing hypotheses, we conducted a genetic–linguistic study of 34 modern Indo-European (IE) populations. In this study, genetic histories of paternal and maternal migrations in these IE populations were elucidated using phylogenetic networks of Y-chromosomal and mitochondrial DNA haplogroups, respectively. Unlike previous studies, we quantitatively characterized the languages based on lexical and phonemic systems separately. We showed that genetic and linguistic distances are significantly correlated with each other and that both are correlated with geographical distances among these populations. However, when controlling for geographical factors, only the correlation between the distances of paternal and lexical characteristics, and between those of maternal and phonemic characteristics, remained. These unbalanced correlations reconciled the two seemingly conflicting hypotheses.

## INTRODUCTION

The hypothesis that language usage follows matrilineal inheritance has been supported by genetic evidence, as in the Austronesian-speaking populations and South American Indians [1,2]. This is called as the mother tongue hypothesis *sensu stricto*. In contrast, on the basis of other findings from genetic and anthropological research [3–9], population geneticists and anthropologists advocate the father tongue hypothesis, which cites that a strong correlation exists between languages and Y-chromosomes. A global picture of sex-specific transmission of language change at the population level has been described by Forster and Renfrew [10]. They summarized that the paternal lines dominate the survivor language in an already-populated region, whereas the maternal lines reflect only the ancient settlement. Therefore, the father tongue hypothesis seems to prevail over the mother tongue hypothesis. However, controversy between these two hypotheses for Indo-European (IE) populations suggests that Y-chromosomal composition in paternal lines may be an essential predictor of language, but not the only one [10].

In addition, quantified language affiliations, such as the designation of language families and subgroups [5], and divergence times deduced from the tree [7], have been used to measure linguistic difference in such studies. However, these two types of data, which can be extracted from linguistic documents, have been argued to be coarse estimations of language differences [11]. Such data provide only holistic evolutionary hints of languages without fully considering linguistic compositions, including lexical and phonemic systems, which may portray distinct evolutionary processes. The evolution of lexical systems, such as the loss or gain of core vocabulary, can trace language divergence [12]. In comparison, the evolution of phonemic systems is more complicated. Phonemes can change not only diachronically but also synchronically, such as via contact-induced (i.e. phoneme borrowings [13]) or spontaneous evolution (i.e. Great Vowel Shift [14]). However, some researchers suggest that in contrast to lexical systems, phonemic systems could be more conservative and provide earlier insights into the evolution of languages [15,16].

## RESULTS

**Figure 1.**
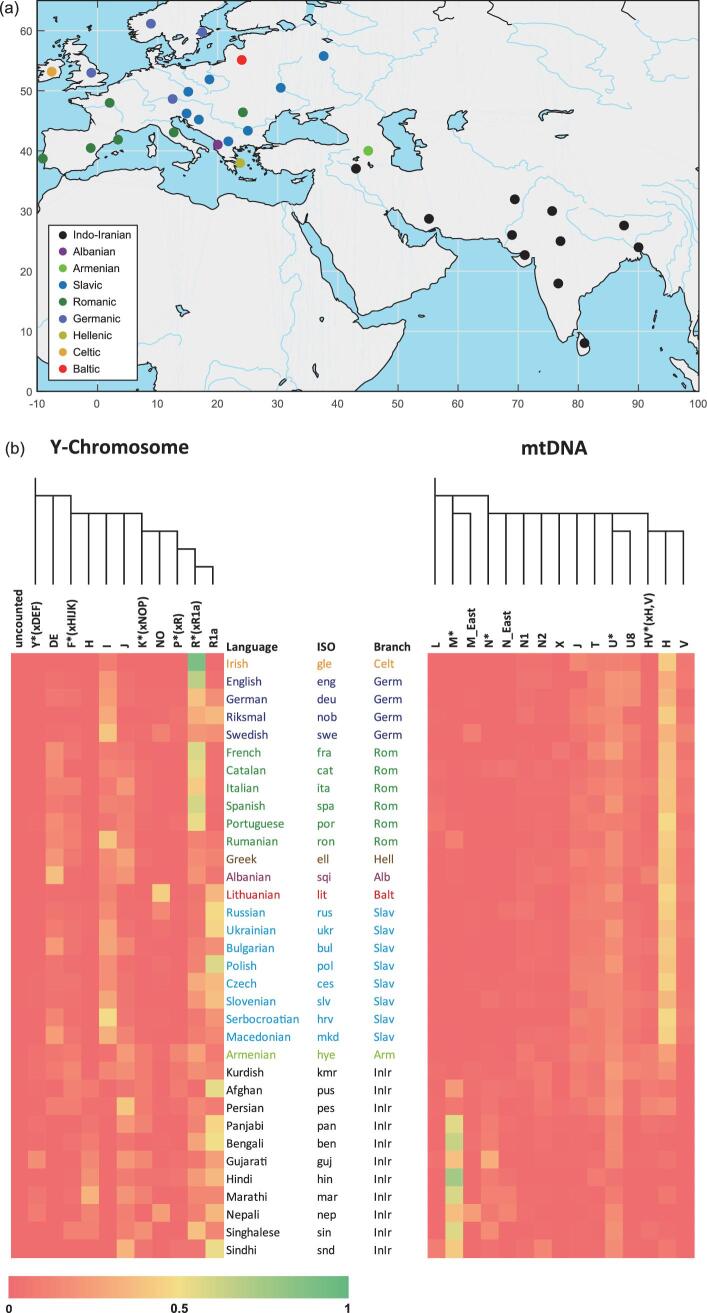
(a) Geographical locations of 34 modern Indo-European populations, coloured by language group. (b) The heat maps of Y-chromosomal and mtDNA haplogroup frequencies of 34 Indo-European populations, aligned with the population speaking each language.

Here, we reassessed the correlation between genetic and linguistic characteristics in 34 modern IE populations (Fig. 1a), for which all four types of data set (lexicon, phonemes, Y-chromosomal composition and mitochondrial DNA (mtDNA) composition) are available. We assembled compositions of the Y-chromosomal and mtDNA haplogroups or paragroups from the corresponding IE populations, which reflect paternal and maternal lines, respectively (see Supplementary section S1.1 and Fig. 1b). These haplogroups or paragroups were defined using stable mutations so that they were all formed already in the Palaeolithic Age (over 10 000 years ago) [17,18]. For example, the categorization of lineages was not changed during the evolutionary processes of IE languages, therefore representing the mixing process of the ancestral populations. Instead of the formerly used linguistic classification or coalescence time, we utilized two types of linguistic data representing distinct evolutionary processes of language systems (see Supplementary section S1.2). The first type was the lexicon of IE languages from Dunn's lexical data set [19], which is publicly available. The other was phonemic data from the PHOIBLE database [20], which contains segment types corresponding to the sound system of the IE languages. Although genetic and linguistic characteristics all reflect the ethnogenetic history of IE population divergence and interaction, they portray different evolutionary processes.

**Figure 2.**
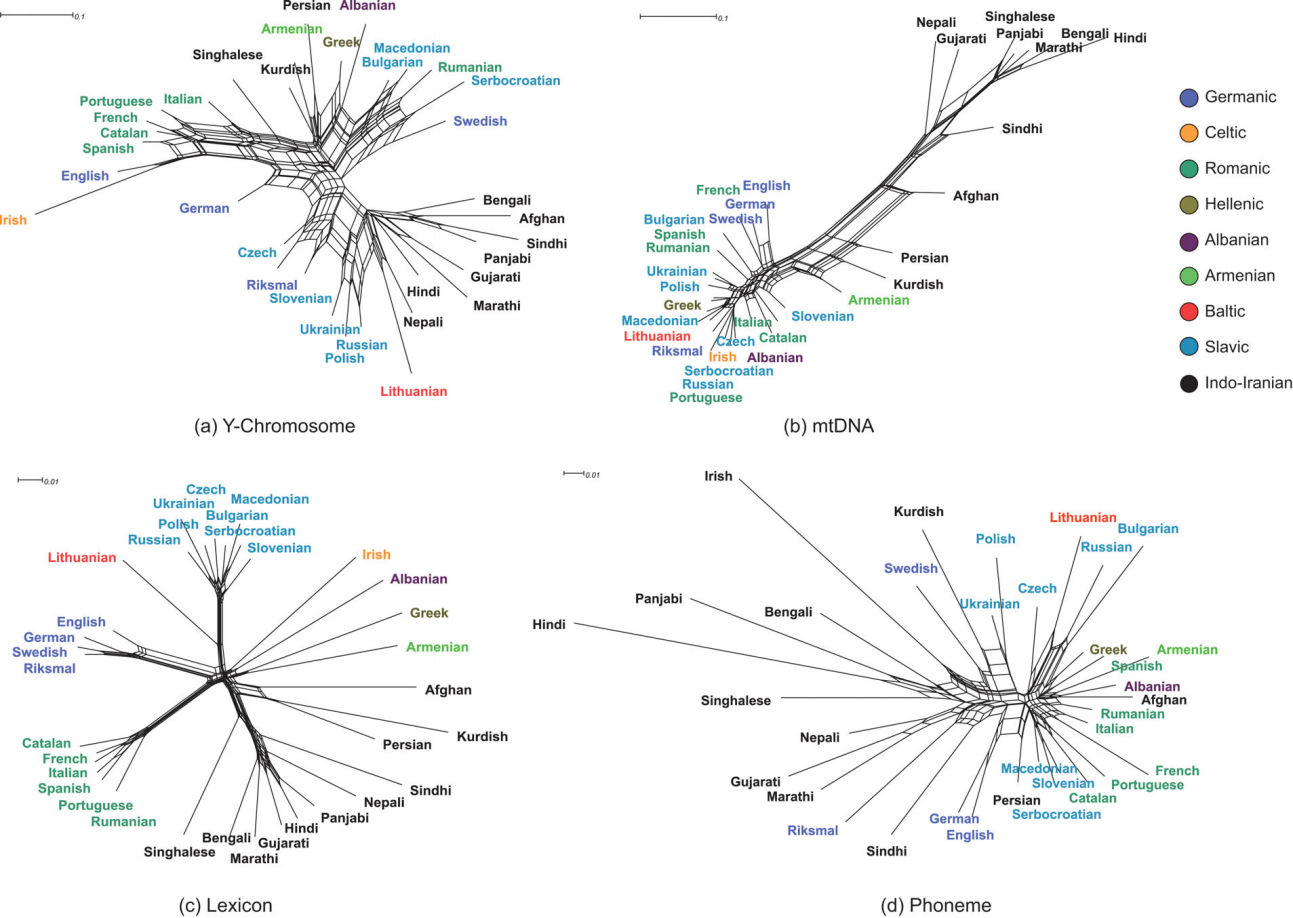
Neighbour-Nets of 34 Indo-European populations calculated from the Euclidean distance matrices using (a) Y-chromosomal haplogroups and (b) mtDNA haplogroups; Neighbour-Nets of IE languages calculated from the Hamming distance matrices using (c) lexicon and (d) phonemes. The colours in the legend correspond to the language groups.

Neighbour-Nets were constructed to delineate the differences between 34 IE population groups clustering at the genetic and linguistic levels (Fig. 2). The reticulations within each net reflect conflicting signals against tree-like structures and support incompatible groupings [21]. These structures are likely produced by potential horizontal transmission between populations or languages such as admixture, and potential parallel evolution in linguistics as well [22]. The Neighbour-Net for Y-chromosomes with substantial reticulations shows complicated relationships among IE populations (Fig. 2a), indicating substantial historical population contact and admixture among the males. In contrast, the Neighbour-Net for mtDNA in Fig. 2b clearly illustrates an East–West geographical polarization, indicating two major IE populations in matrilineages: Indo-Iranian and European. Due to the limited lexical borrowings in Dunn's lexical data set [12], the Neighbour-Net for lexicon thus appears to better approximate a tree-like structure with fewer reticulations than the phonemic Neighbour-Net. The clustering groups for languages based on lexicon were consistent with traditional linguistic classifications. In contrast, the Neighbour-Net for phonemic systems showed evidence of a substantial conflicting signal between phonemic characteristics. The network did not accurately recover many attested phylogenetic relationships among IE languages. None of the language groups were monophyletic at the phonemic level.

**Figure 3.**
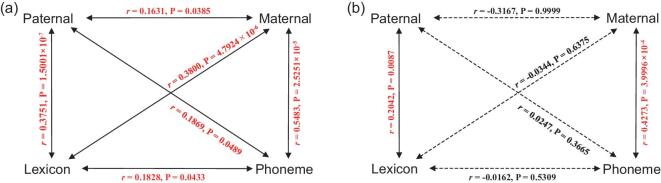
Mantel correlations between four distance matrices for Y-chromosome, mtDNA, phoneme and lexicon. (a) Mantel correlations and (b) partial Mantel correlations when controlling for geographical effects. The number of permutations of the Mantel test was set at 10 000. The red text shows significant Mantel correlations. Solid lines represent a *P*-value < 0.05. Dashed lines represent no significance, *P*-value > 0.05.

To investigate the relationships between genetic and linguistic characteristics, we performed the Mantel test on the pairwise genetic and linguistic distance matrices of 34 IE populations. Fig. 3a clearly shows that the genetic and linguistic characteristics were strongly correlated with each other. However, these correlations have been argued to be false signals because all these variables could be dependent on geography [23]. In 34 IE populations, all the genetic and linguistic distances indeed had significantly positive relationships with the geographical distances for these IE populations (see Supplementary section S2.1).

To exclude the geographical effects, we then adopted the partial Mantel test to reappraise the relationships between genetics and linguistics in these populations (Fig. 3b). When controlling for the effect of geographical distance of pairwise IE populations, there was no significant correlation between Y-chromosomal and mtDNA distance matrices. It indicated that paternal and maternal lineages had different ethnic histories in IE populations. Similarly, lexical and phonemic systems of IE languages experienced different evolutionary processes because of no correlation between lexical and phonemic distances. In particular, the correlations between the Y-chromosomal and phonemic distance matrices, as well as those between the mtDNA and lexical matrices, were no longer significant. This result therefore suggests that both Y-chromosome–phoneme and mtDNA–lexicon relationships between the IE samples could be sufficiently predicted by their geographical distance. However, the correlation between Y-chromosomal and lexical distances remained significant (partial Mantel r = 0.2042, *P*-value < 10^−3^), as did the correlation between mtDNA and phonemic distances (r = 0.4273, *P*-value < 10^−3^). In addition, we performed two alternative partial statistical tests to validate the reliability of these observations (see Methods). The results of three partial statistical tests were consistent with each other (Table S1). Such observations of unbalanced correlations, after removing the effect of geography, suggest that the change in lexicon reflects the differences in paternal lines, while phonemic dissimilarity reflects the differences in maternal lines. Moreover, we adopted an alternative lexical data set provided by Bouckaert *et al.* [24] to validate the statistical results of Mantel and partial Mantel tests, especially for the correlation between Y-chromosomes and lexicon (see Supplementary sections S1.2 and S2.2). The results obtained from this lexical data set were consistent with those for Dunn's data set. In addition, the Jackknife resampling approach was used to evaluate the robustness of the correlation between genetics and linguistics (see Supplementary section S2.3, and Tables S2 and S3).

These observations of unbalanced correlation between genetics and linguistics could be explained by population contact and admixture at first. If there is no contact and admixture between the populations or languages, the phylogenies of genetics and linguistics should ideally follow tree-like structures and resemble each other. However, population contacts have long been known to change local population structures and language systems. The causes of such population contacts include marriage between neighbouring populations or between local people and immigrants, such as military conquerors or merchants. In particular, the different performances of female and male dispersal have confirmed that females live more locally than males [25–28] (see Supplemantary section S2.4). In other words, the immigrants tend to be highly sex-biased with a higher concentration of males [10,29]. This could be also why we found no significant correlation between paternal and maternal lines in IE populations, when controlling the geographical effects. When immigration is associated with social prestige such as colonists, the immigrants form a new community that speaks the languages brought with them, while their spouses (usually women) are from the local region. Therefore, the social prestige of male immigrants could reasonably lead to the correlation between the Y-chromosome and languages [30].

The language learning by local women could constitute the reason for the unbalanced correlation of mtDNA to lexicon and phonemes. Due to the social prestige of male immigrants, their local spouses have to adopt the language of their husbands and pass it to future generations [6,10,15]. This process is second language acquisition and easily develops language fossilization [31]. The language fossilization is a linguistic mechanism that sees a learner of a second language tend to preserve some linguistic features of the first language, and develop a form of inter-language [31]. Under such circumstances, women can easily replace the lexicon from another language [21], but attempt to retain local accents influenced by their native language [32]. In other words, women change to adopt the same word usage as their husbands in daily life but still speak using their own pronunciation. In mixed-language marriages with these male immigrants, women prefer to pass down their inter-languages to offspring [10,33]. As a result, we get the correlation between mtDNA and phonemes that we observed. Hence, we courageously propose a hypothetical scenario for IE populations where the lexical system of language is dominated by their father, while the phonemic system of language is determined by their mother.

The co-evolution between genes and languages is asymmetrical in IE populations. Our findings provide strong statistical evidence to reconcile the conflicting father tongue and mother tongue hypotheses. The populations involved in this study are located within a single continent and all of them speak languages belonging to the IE language family. Therefore, much of the genetic pattern may have its roots in the spread of IE languages. Further cross-continental comparison between genetic and linguistic data would provide us with more remarkable co-evolutionary processes of population and language. Notably, what we observed from the correlation between linguistics and genetics is macroscopic. The scenario that the mother learns her husband's language and teaches the children is definitely one possible mechanism, which has been elaborated by historical linguist van Driem [30]. In the future, more detailed exploration is warranted into the mechanisms of language change at the micro level, including infants’ language acquisition and development from the father and mother, and even other social structures. Moreover, the present research paradigm can be extended to other human cultural and social traits [34–36]. On basis of interdisciplinary approaches, there is an important challenge for us to re-examine several general hypotheses of population and cultural evolution at the global scale.

## METHODS

### Distance matrices and Neighbour-Net

To delineate the relationships between 34 IE populations and their languages, we applied the Neighbour-Net method [37,38] to the four data sets of genetic and linguistic properties, respectively. The genetic Neighbour-Nets were calculated from distance matrices on haplogroup frequencies using the Euclidean distance method. According to the linguistic distance matrices used in Creanza *et al*. [13], we applied Hamming distance matrices [39] to comparing the presence/absence of traits (lexicons and phonemes). Notably, for the Bouckaert data set, each hamming distance of pairwise languages was calculated by ignoring all missing cognate sets in pairwise languages compared. The linguistic Neighbour-Nets were established with Hamming distance matrices from lexical and phonemic data. In addition, we applied the orthodromic distance (great circle distance) of two locations for the metric of geographical distance, and transformed the distance (d) into the logarithmic scale following the formula log10(d). The hamming distance for the Bouckaert data set and geographical distance calculation was implemented in Matlab. All network analyses were performed in SplitsTree4 (http://www.splitstree.org/) using default settings.

### Mantel test and partial Mantel test

In this paper, we used the Mantel test to detect the relationships between languages and genes, and the partial Mantel test to further study the correlation between languages and genes controlled with geographical effects. All statistical tests were implemented in Matlab® R2015b (MathWorks, Inc.). The Matlab scripts for the Mantel test and partial Mantel test were provided by Prunier *et al*. [40] (URL: http://www.jeromeprunier.eg2.fr/5.html).

To validate the credibility of the statistical results, we adopted two alternative partial correlation tests. The first was the linear Pearson's correlation test [41] implemented in Matlab® as the function ‘partialcorr’. The other was a modified partial Mantel test, which was developed by Smouse *et al*. [42], to examine the Mantel correlation between two residuals from linear regressions of genes/languages on geographical distance metrics, respectively. Specifically, we designated the three matrices to be compared as A, B and C. The users tested the significance of partial correlation by computing residual matrices from the regressions of A on C and B on C, and then carried out a Mantel test between the two residual matrices with the permutation approach. In this process, we performed the Matlab script of the Mantel test programmed by Enrico Glerean (http://becs.aalto.fi/∼eglerean/permutations.html). The numbers of permutations in all Mantel or partial Mantel tests were set at 10 000 in this study.

### Principal component analysis and Procrustes analysis

We here conducted a series of principal component (PC) analyses [43] (PCA) to identify the principal coordinates of the high-dimensional linguistic or genetic data of IE populations. Then, we performed Procrustes analysis of each genetic and linguistic PC versus the geographical coordinates of these IE populations. The rationale of Procrustes analysis [44,45] is to find an optimal transformation of two or more maps that maximize the similarity of the transformed maps, and to score the similarity between two optimally transformed maps. In this study, the two maps being compared are the two-dimensional plot of the first two PCs, and the geographical map of the latitudes and longitudes of 34 IE populations. A permutation test [46,47] can then measure the probability that a randomly chosen permutation of the points in any one map produces a greater similarity score than that observed for the actual points in the other map.

Following Wang *et al*. [48], we calculated a similarity score on the statistic ${t_0} = {\rm{\ }}\sqrt {( {1 - D} )} $, where D is the minimized sum of squared distances in Procrustes analysis. We then calculated empirical *P*-values for *t*_0_ values over 100,000 permutations of geographical locations. All computational procedures of PCA, Procrustes analysis and permutation tests were implemented in Matlab® R2015b (MathWorks, Inc.).

### Jackknife resampling method

We performed the Jackknife resampling approach to evaluate the robustness of the statistical conclusions based on a partial Mantel test. In this study, we considered the balance of the samples sizes between Indo-Iranian and European populations, and designed two schemes of Jackknife resampling approach [49–51]:

Scheme I: we sampled all the available Indo-Iranian populations from the data set and randomly selected equal amounts of populations from the total European populations.

Scheme II: we randomly selected the same number of population samples from the total IE populations in order to compare to the resampling in scheme I.

Accordingly, we resampled 22 IE populations (11 Indo-Iranian + 11 European for scheme I, and randomly 22 out of 34 in scheme II) for Dunn's data set, and 18 (9 + 9 for scheme I, and 18/32 for scheme II) for a new lexical data set of 207 words by Bouckaert *et al*. For each resampling scheme, the random selection was repeated for 500 times, and thus 500 Jackknife-resampled data sets of selected population sample were generated. For each data set, we reconducted partial Mantel tests to examine the correlation between these genetic and linguistic data controlling for geographical effects (Y-chromosome and lexicon, Y-chromosome and phoneme, mtDNA and lexicon, and mtDNA and phoneme). The correlation coefficients and *P*-values were recalculated. For the correlation coefficients obtained via the Jackknife method, we listed the statistical descriptions including the median, minimum, maximum and 95% confidence intervals in Table S2. For the distribution of *P*-values, we calculated quantiles (0.25, 0.50 and 0.75) and counted the number of *P*-values less than 0.05 or 0.01. We counted the occurrence of *P*-value < 0.05 and < 0.01 out of Jackknife 500 replicates to measure the robustness. Notably, the occurrence was a relative value to compare the results of different partial Mantel tests.

### Data availability

All linguistic and genetic data that support the findings of this study are available within the paper and its supplementary information files.

## Supplementary Material

Supplemental Files
