# Supplementary material for: Reconciling the father tongue and mother tongue hypotheses in Indo-European populations
Source: Natl Sci Rev. 2018 Aug 20;6(2):293–300. doi: 10.1093/nsr/nwy083 (PMC8291526; doi:10.1093/nsr/nwy083)
Supplement: Supplemental Files [file nwy083_supplemental_files.zip › Revised SI.docx]

**Supplementary Information**

**Reconciling the father tongue and mother tongue hypotheses in Indo-European populations**

Menghan Zhang, Hong-Xiang Zheng, Shi Yan, Li Jin

**Sections**

**S1. Materials**

**S1.1. Collection of genetic data**

**S1.2. Preparation for linguistic data**

**S1.3. Intersection set of genetic and linguistic datasets**

**S2. Supplementary Text**

**S2.1. Relationships between genetic and geographic distances, and between linguistic and geographic distances among IE populations**

**S2.2. Validation on correlations between genetics and linguistics using Bouckaert’s lexical dataset**

**S2.3. The robustness of statistical results on the correlations between genetic and linguistic data**

**S2.4. Female and male dispersal in Indo-European population**

**S3. References**

**Figure S1**

**Table S1**

**Extended Data Table S2-S9**

**S1. Materials:**

**S1.1 Collection of genetic data**

We collected more than 50 references containing Y chromosomal haplogroup frequency data of Indo-European languages. To compare the data using different tested SNP (single nucleotide polymorphism) sets, we manually binned the samples into 11 haplogroups or paragroups: DE, F*(xHIJK), H, I, J, K*(xNOP), NO, P*(xR), R*(xR1a), and Y*(xDEF) (Table S3). As indicated by most of the related literatures, above classification could show essential variations among Indo-European populations. Finally, we retained the Y-haplogroup frequency data from 37 references, with each population having at least 20 genotyped samples. The remaining references were discarded for we could not achieve a similar resolution of haplogroup classification.

Similarly, from the public literature using haplogroup-unbiased sampling, we binned the samples with either full sequences or hyper-variant region sequences (HVR) of mitochondrial DNA (mtDNA) data into 15 haplogroups or paragroups (Table S4).The 15 haplogroups or paragroups were L, M, M_East, N, N_East, N1, N2, HV, H, V, J, T, U, U8 and X, of which N1 contained I, N2 contained W and U8 contained K. Generally, our classification was based on the traditional haplogroup assignment for West Eurasian mtDNA gene pool. Specifically, M_East included the haplogroups common in East Asia such as M7, M8, M9, D, and M12’G, while M included other M lineages in West Eurasia and South Asia, such as M1, M2, and M5. Similarly, N_East consisted of the haplogroups common in East Asia such as N9, A, B and R9 while N consisted of the rare haplogroups present in West Eurasia and South Asia, such as R2 and R30. Finally, we collected frequency data for IE populations from 40 references, with population sizes of at least 25 and an average of 177.

We kept Y-chromosomal data for 9,977 male samples from 28 references and mtDNA data for 6,033 samples from 40 references. The paternal and maternal compositions under the haplogroups or paragroups noted above were also calculated.

**S1.2** **Preparation of linguistic data**

The linguistic data used to build language phylogenetic trees or networks were compiled from one world-wide phoneme database and two different datasets of basic vocabulary.

For phonemic dataset, we extracted phonemes (or segment types) and geographic coordinates for these IE language populations from PHOIBLE (PHOnetics Information Base and Lexicon)[1] (see Table S6 and S7). PHOIBLE is a linguistic database with 2,155 inventories that contains 2,160 phonemes (or segment types) found in 1,672 distinct languages in the world, and contains ISO 639-3 codes and geographic coordinates. A form of this database is also available at: http://phoible.org/. Notably, phonemes used as segment types here include consonants, vowels and tones. Therefore, we did not distinguish between the names of “phoneme” and “segment type”. In the intersection set of genetic and linguistic datasets, there were total 353 binary coded phonemes as indicating the presence/absence in IE languages.

In addition, two lexical datasets consisted of two publicly available cognate datasets. The first one is from the published dataset of 101 IE languages used in Dunn *et al.*[2] (hereafter Dunn dataset). The dataset depends on the Comparative Indo-European Lexical Database (IELex) compiled by Dyen[3] and an expanded version of the data described in Gray and Atkinson[4]. The dataset consisted of total of 2,301 cognate sets on the basis of the Swadesh 100 word-list[5, 6]. As noted in Dunn *et al.*[2], the cognates were identified by expert linguists, validated according to etymological dictionaries, and stripped of potential lexical borrowings. The detailed descriptions of the cognates, such as lexical meaning and phonological form, are listed in the IELex database (website available: http://ielex.mpi.nl/). The dataset have been encoded into binary characters representing the presence (“1”) or absence (“0”) of each cognate set in each Indo-European language[4]. Based on this binary coding dataset intersected with genetic and phonemic datasets, we eliminated the cognate sets that were all present, absent and all missing, absent in 34 IE languages. Finally, we obtained 1,186 cognate sets (see Table S5) as lexical data of 34 IE languages.

To validate our statistical results of correlations between genetic components (Y-chromosome, mtDNA) and lexicon, we adopted alternative lexical dataset, which was from the supplementary materials of Bouckaert *et al.*[2] (hereafter Bouckaert dataset). This dataset were on the basis of the IELex database[3] and other linguistic literatures listed in their paper[7]. Compared to Dunn dataset across Swadesh 100 word list, the word forms in Bouckaert *et al*. were recorded across 207 meanings and also made cognate judgments by expert linguistics in 103 IE languages including contemporary and ancient languages. These meanings were basic vocabulary items – including kinship terms (i.e. mother, father), body parts (i.e. hand, foot), terms for the natural world (i.e. sun, moon, and water) and basic verbs (i.e. to run, to fly, and to come). The cognate judgement for this dataset has been conducted by expert linguists. Similar to the data process for Dunn dataset, 6,280 cognate sets have been coded as binary characters showing the presence or absence of each cognate set in a language. Intersected by other three datasets (phonemes, Y-chromosome and mtDNA), there were 33 contemporary Indo-European languages in Bouckaert dataset matched with ones in Dunn dataset. Next, we eliminated the cognate sets that were all present, absent and all missing in this dataset of 33 IE languages. Accordingly, 5,305 cognate sets were retained. In addition, we had to exclude Kurdish language due to over 70% missing data in all lexical cognate sets of this Indo-Iranian language. Finally, we obtained a usable dataset of 5,305 cognate sets in 32 IE languages intersected with phonemic and genetic dataset.

Nelson-Sathi *et al*. pointed out that Dunn dataset includes no more than 5% known horizontal influence (i.e. word borrowings) among the IE languages[8]. In contrast to Dunn dataset, Bouckaert dataset could include more horizontal influence among IE languages, due to its larger inventory of basic vocabulary.

**S1.3 Intersection set of genetic and linguistic datasets**

To compare four types of datasets (Y-chromosomes, mitochondria, phonemes, and lexicon), we retained the intersection set of Indo-European-speaking populations as for the following analyses. There were 34 IE populations/languages in the intersection set of Y-chromosome, mtDNA, phonemes, and Dunn’s lexical datasets. Meanwhile, there were 32 available IE populations/languages in the intersection set of Y-chromosome, mtDNA, phonemes, and Bouckaert’s lexical datasets.

**Supplementary Text:**

**S2.1** **Relationships between genetic and geographic distances, and between linguistic and geographic distances among IE populations**

We first performed Mantel test to examine the relationships between genetic/linguistic and geographic distances among 34 IE populations (Y-chromosome: Mantel r = 0.4282, *p*-value < 10^-5^; mtDNA: r = 0.7904, *p*-value < 10^-5^; Lexicon: r = 0.5038, *p*-value < 10^-5^; Phoneme: r = 0.3885, *p*-value < 10^-5^). On the other hand, we quantified broad similarities between geographic locations of IE populations and the first two PCs of genetic and linguistic data obtained via Principal Component Analysis[9], along with Procrustes Analyses[10] (see Supplementary S1.5). The first two principal components of Y-chromosome together explained 68.18% of the total variations in Y-chromosomal data; the first two PCs for mtDNA data explained 90.25% of the total variations; the first two PCs for lexicon explained 29.73% of the total variation for Dunn dataset; and the first two PCs for phonemic data explained 22.29% of the total variations. There was significant concordances between the first two PCs of genetic data and geographic locations for these Indo-European population (Y-chromosome: Procrustes *t_0_* = 0.7647, *p*-value<10^−5^; mtDNA: *t_0_* = 0.9692, *p*-value<10^−5^), as well as those between linguistic data and geographic locations (Lexicon: *t_0_* = 0.8460, *p*-value<10^−5^; Phoneme: *t_0_* = 0.6824, *p*-value<10^−5^).

Following the arguments of Rosser *et al.*[11], we next performed Spearman’s correlation analyses to validate these statistical results. We calculated Spearman’s rho values (ρ) and corresponding *p*-values for the association between pairwise distance metrics for geography and pairwise Euclidean distance metrics for Y-chromosome and mtDNA; and pairwise Hamming distance metrics for lexicons and phonemes. The geographic distances were scaled down by the base 10 logarithm. Our results showed that all genetic and linguistic properties had significantly positive relationships with geography (Y-chromosome: Spearman’s rho = 0.4262, *p*-value = 0.00; mtDNA: rho = 0.8004, *p*-value = 0.00; Lexicon: rho = 0.4660, *p*-value = 1.3973×10^-31^; Phoneme: rho = 9.0560×10^-27^).

**S2.2** **Validation on correlations between genetic characteristics and lexicon of 32 IE populations based on Bouckaert dataset**

For Bouckaert lexical dataset, all statistical results of Mantel and partial Mantel tests were consistent with those for Dunn dataset. In particular, the significant correlation between lexical and geographic distances among 32 IE populations was observed for Bouckaert dataset (Mantel r = 0.5527, *p-*value < 10^-5^; Spearman ρ = 0.5597, *p*-value = 3.1032×10^-42^). Moreover, we found that lexical distances were correlated with both Y-chromosomal and mtDNA distances among 32 IE languages (lexicon-Y chromosome: Mantel r = 0.4501, *p-*value < 10^-5^; lexicon-mtDNA: r = 0.4137, *p-*value < 10^-5^). When controlled for the effects of geography in partial Mantel test, the significant correlation between Y-chromosome and lexicon was still retained (partial Mantel r = 0.2887, *p-*value < 10^-5^), but not between mtDNA and lexicon (partial Mantel r = -0.0500, *p*-value = 0.7002). These observations were consistency with those for Dunn dataset, still supporting the Father Tongue Hypothesis at lexical level.

**S2.3 The robustness of statistical results on the correlations between genetic and linguistic data**

In our dataset, the sample sizes of Indo-Iranian and European populations were unbalanced, and the geographic distribution of these Indo-Iranian and European populations were heterogeneous. According to Guillot and Rousset’s statements, found that statistical power of partial Mantel test is sensitive to sample size and the heterogeneous spatial distributions of samples[12]. Therefore, we adopted Jackknife resampling approach to evaluate the robustness of the correlation between genetic and linguistic distance matrices based on classic partial Mantel test and the partial Mantel test by Smouse *et al.*

As shown in Table S2, for Dunn dataset, the non-significant correlations between Y-chromosome and phonemes, and between mtDNA and lexicon are all reliable, due to 5.2% occurrences of *p*-value<0.05 out of 500 jackknife replicates in both Scheme I and II. Similarly, the robustness of significant correlation between mtDNA and phonemes was great in both jackknife Scheme I and II, based on whether using classic Mantel test or the Mantel test by Smouse *et al.* The occurrences of *p*-value<0.05 were all over 400 out of 500 jackknife replicates. This reliable correlation supported the Mother Tongue hypothesis.

For the correlation between Y-chromosome and lexicon in Dunn lexical dataset, different resampling strategies of the Jackknife Scheme I and II led to the difference that the maximum occurrence of *p*-values<0.05 was 123 in Scheme I, while the maximum one in Scheme II was 278. In contrast to the correlation between mtDNA and phonemes, the reliability of correlation between Y-chromosome and lexicon seems limited. In our opinion, this is likely attributed to limited word borrowings (~ 5% of the words) involved in Dunn database[8]. Due to this, the cognate data might under-represent those information introduced through admixture, and reduce the correlation between Y-chromosome and lexicon, especially when we adopted various jackknife resampling strategies. Even though, since the correlation between language classification (well represented through the cognate data as shown in Fig. 1c) and Y-chromosome have been sufficiently supported by multiple previous studies, the correlation between mtDNA and pronunciation is robust to support our discovery.

For Bouckaert dataset, the correlation between Y-chromosome and lexicon was more reliable than that for Dunn dataset. As shown in Table S3, the occurrence of partial Mantel *p*-values<0.05 was 398 in Scheme I and 431 in Scheme II out of 500 Jackknife resampling replicates. These results were likely attributed to the larger word-list of Bouckaert dataset reflecting more detailed evolutionary information of IE languages than Dunn dataset. Such robust correlation supported the Father Tongue Hypothesis at lexical level. In addition, the correlation between mtDNA and phonemes controlled for the geographic effects remained significant whether in Scheme I or II, supporting the Mother Tongue Hypothesis at phonemic level.

**S2.4 Female and male dispersal in Indo-European population**

The performances of female and male dispersal are different. In contrast to the older genetic work (such as [13]), male dispersal is however greater than female in some regions such as the central Africa [14], the Caucasus [15] and China [16]. Lippold *et al.* [17] found that the metric of Fixation index (Fst) for mtDNA of worldwide populations was primarily higher than Y-chromosome, and concluded that female could live more locally than male. To assess the different performance of female and male dispersal, we applied the same approach mentioned in [13] on our genetic data of Indo-European populations. We modeled the regression of genetic distance onto geographic distance, and found that the slope for the mtDNA data (0.57231) was higher than the Y-chromosome data (0.20599) of IE populations. It indicated a more rapid increase of genetic distance for the mtDNA (female) than Y-chromosome (male). The population dispersal in a specific region could efficiently decrease genetic distances among the populations settled in this region. In contrast, low population contact such as geographic isolation could increase the genetic distance among the populations. Therefore, in our study, it is reasonable to believe that male dispersal is greater than female in IE regions. In addition, this result could be valid under patrilocal society and peaceful period in our opinion.

**Reference:**

[1] S. Moran, D. McCloy, and R. Wright. PHOIBLE online. *Leipzig: Max Planck Institute for Evolutionary Anthropology,* 2014.

[2] M. Dunn, S. J. Greenhill, S. C. Levinson, and R. D. Gray. Evolved structure of language shows lineage-specific trends in word-order universals. *Nature.* 2011 ; 473:79-82.

[3] I. Dyen and P. Black. An indo-european classification: a lexicosta-tistical experiment. *Trans Am Phil Soc.* 1992; 82, p. 825,.

[4] R. D. Gray and Q. D. Atkinson. Language-tree divergence times support the Anatolian theory of Indo-European origin. *Nature,* 2003; 426:435-439.

[5] M. Swadesh. Lexico-statistic dating of prehistoric ethnic contacts: with special reference to North American Indians and Eskimos. *Proc Am phil soci.* 1952; 96:452-463.

[6] M. Swadesh, *The origin and diversification of language*: Transaction Publishers, 1971.

[7] R. Bouckaert, P. Lemey, M. Dunn, S. J. Greenhill, A. V. Alekseyenko, A. J. Drummond*, et al.*. Mapping the origins and expansion of the Indo-European language family. *Science,* 2012; 337:957-960.

[8] S. Nelson-Sathi, J.-M. List, H. Geisler, *et al.*. Networks uncover hidden lexical borrowing in Indo-European language evolution. *Proc Biol Sci.* 2011 ; 278:1794-1803.

[9] Jolliffe and Ian. Principal Component Analysis. *Springer Berlin.* 1986; 87:41-64.

[10] C. Wang, Z. A. Szpiech, J. H. Degnan, M. Jakobsson, T. J. Pemberton, J. A. Hardy*, et al.*. Comparing spatial maps of human population-genetic variation using Procrustes analysis. *Stat Appl Gen & Mol Bio,* 2010; 9: Article 13,.

[11] Z. H. Rosser, T. Zerjal, M. E. Hurles, M. Adojaan, D. Alavantic, A. Amorim*, et al.*. Y-Chromosomal Diversity in Europe Is Clinal and Influenced Primarily by Geography, Rather than by Language. *Am J Hum Genet,* 2000; 67:1526-43.

[12] G. Guillot and F. Rousset. Dismantling Mantel tests. *Methods in Ecology & Evolution.* 2011; 4:336–344.

[13] Seielstad M T, Minch E, Cavalli-Sforza L L. Genetic evidence for a higher female migration rate in humans. *Nat Genet*. 1998; 20(3): 278-80.

[14] CAVALLI‐SFORZA, L. L., & Hewlett, B. Exploration and mating range in African Pygmies. *Ann Hum Genet*. 1982; 46(3): 257-270.

[15] Nasidze I, Ling E Y S, Quinque D, et al. Mitochondrial DNA and Y-chromosome variation in the Caucasus. *Ann Hum Genet*. 2004; 68(3): 205-221.

[16] Wen B, Li H, Lu D, et al. Genetic evidence supports demic diffusion of Han culture. *Nature*. 2004; 431(7006): 302-305.

[17] Lippold S, Xu H, Ko A, et al. Human paternal and maternal demographic histories: insights from high-resolution Y chromosome and mtDNA sequences. *Investig Genet*. 2014; 5(1): 13.

**Table S1.** The comparison results of partial correlation coefficients and *p*-values (in parentheses) when controlling geographic distance. The shaded value represents *p*-value < 0.05.

| **Controlled for geographic effects** | **Classic Partial Mantel Test** | **Partial Mantel Test by Smouse *et al.*** | **Partial Linear Pearson Correlation** |
| --- | --- | --- | --- |
| **Phoneme - Y Chromosome** | 0.0247 (0.3665) | -0.0733 (0.7708) | 0.0247 (0.5604) |
| **Lexicon - Y Chromosome** | **0.2042 (0.0087)** | **0.1856 (0.0185)** | **0.2042 (1.1056×10^-6^)** |
| **Phoneme - mtDNA** | **0.4273 (3.9996×10^-4^)** | **0.3831 (4.8177×10^-4^)** | **0.4273 (2.9435×10^-26^)** |
| **Lexicon - mtDNA** | -0.0344 (0.6375) | -0.1323 (0.9137) | -0.0344 (0.4172) |

**Extended Table S2**

Table Description: The statistical descriptions of partial Mantel test and the partial Mantel test by Smouse *et al.* using Dunn dataset in Jackknife Scheme I and II.

**Extended Table S3**

Table Description: The statistical descriptions of partial Mantel test and the partial Mantel test by Smouse *et al.* using Bouckaert dataset in Jackknife Scheme I and II.

**Extended Data Table S4**

Table Description: Data of 34 contemporary Indo-European population for Y-Chromosomal and corresponding frequency distributions.

**Extended Data Table S5**

Table Description: Data of 34 contemporary Indo-European population for mtDNA haplogroups and corresponding frequency distributions.

**Extended Data Table S6**

Table Description: Phonemic data of 34 contemporary Indo-European population for binary-coded phonemes occurred in all these corresponding languages.

**Extended Data Table S7**

Table Description: Dunn lexical dataset of 34 contemporary Indo-European population for corresponding binary-coded lexical cognates.

**Extended Data Table S8**

Table Description: Bouckaert lexical dataset of 32 contemporary Indo-European population for corresponding binary-coded lexical cognates.

**Extended Data Table S9**

Table Description: Geographic coordinates of 34 contemporary Indo-European population.
